# Regional variations and socioeconomic factors influencing sex hormone profiles in adolescent girls in Ghana

**DOI:** 10.3389/frph.2025.1579942

**Published:** 2025-11-10

**Authors:** Eric Kyei-Baafour, Sawudatu Zakariah-Akoto, Michael Ofori, Lutherodt Bentum-Ennin, Oscar Nii Otto Darko, Godfred Egbi, Benjamin Abuaku, Collins Ahorlu, Dorothy Yeboah-Manu

**Affiliations:** 1 Immunology Department, Noguchi Memorial Institute for Medical Research, University of Ghana, Accra, Ghana; 2 Nutrition Department, Noguchi Memorial Institute for Medical Research, University of Ghana, Accra, Ghana; 3 Epidemiology Department, Noguchi Memorial Institute for Medical Research, University of Ghana, Accra, Ghana; 4 Bacteriology Department, Noguchi Memorial Institute for Medical Research, University of Ghana, Accra, Ghana

**Keywords:** adolescent girls, estrogen, progesterone, androgen, Ghana

## Abstract

**Introduction:**

Adolescence is a critical period of hormonal changes that affect growth, development, and behaviour. Estrogen, progesterone, and androgen are sex hormones that play important roles in reproductive development and functions. Factors including diet, Health care, and genetic variation, affect hormone production/levels. However, the effect of different environments has not been explored in detail. This study aimed to evaluate hormonal levels in adolescent girls from two regions in Ghana and also assess associated socioeconomic status.

**Method:**

One hundred and sixteen (116) blood samples were drawn from in-school adolescent girls aged 10–19 years who were involved in a qualitative study to explore adolescent girls’ knowledge, perceptions and experiences of hormonal imbalance in Northern and Southern Ghana between June and October 2022. Their hormonal levels were assessed in respect of androgen, estrogen and progesterone to determine the influence of environmental and parents’ socio-economic factors. Using the Enzyme-Linked Immunosorbent Assay (ELISA), serum levels were determined.

**Results:**

Generally, average serum levels of estrogen, androgen, and progesterone were 195.5 (29.2–899.2.0) pg/ml, 60.7 (8.2–687.3) pg/ml, 46.0 (5.2–130.6) ng/ml respectively. When categorized by location, serum estrogen level was 195.8 (35.7–899.2) pg/ml in the north compared to 195.3 (29.2–899.2) pg/ml, in the south, *p* = 0.63. Androgen was 52.8 (8.2–687.3) pg/ml vs. 61.2 (16.0–683.33) pg/ml, *p* = 0.81, and progesterone level was 53.3 (9.2–130.6) ng/ml vs. 43.0 (5.2–111.3) ng/ml, *p* = 0.0019. Northern participants whose mothers did not have any formal education had higher androgen (*p* = 0.009) and estrogen (*p* = 0.0012) levels compared to those from the south. Also, girls with educated fathers had higher progesterone levels (*p* = 0.03). The proportion of parental unemployment was low across locations. Other covariates did not influence hormonal levels (*p* > 0.05).

**Conclusion:**

This study provides useful information on the hormonal profiles of adolescent girls in Ghana which can inform the School Health Education Programme's (SHEP) intervention activities related to reproductive health issues. The study shows that there were some variations in the levels of serum progesterone between the two locations with participants from Northern Ghana having higher levels. It also highlights the need for practices that address the differences in hormonal levels among adolescent girls based on the educational levels of mothers, recognizing its potential implications for their health, fertility, and well-being.

## Introduction

Adolescence (10–19 years) (1), constitutes a pivotal phase of human development, characterized by rapid biological, psychological, and social transformations (2, 3). Central to this process are changes in sex hormone profiles, which play a fundamental role in shaping both physical and psychological well-being (4, 5). The hormones estrogen, androgen and progesterone are an essential hormones for female pubertal development, regulating reproductive maturation, growth, and psychosocial well-being of adolescent girls (6, 7). These hormones are largely regulated by the hypothalamic-pituitary-gonadal (HPG) axis. However, their levels are influenced by environmental, genetic, diatery and socioeconomic factors (8–10).

Socioeconomic factors, diets, access to healthcare services, geographical location, genetic variation and, other environmental factors such as light and temperature drive hormonal levels. Some key indicators of socioeconomic status (SES), such as parental education, family structure, employment status, financial stability (10), have been shown to associate with alterations in sex hormone profiles. Educational and income levels may influence lifestyle including dietery choices which may in turn influence hormonal levels (2, 11–13). Studies have found low education and income to be associated with unhealthy dietary habits, including consuming less balanced meals and skipping breakfast (10, 14). In contrast, higher educational attainment or occupational position (an indicator of higher income) is likely to result in the maintenance of a healthy diet (15). These factors are important in the context of Ghana with a diverse population covering different ethnicities with a diverse socio-economic structure (14–16).

A vicious cycle has also been reported between healthcare services and socioeconomic factors. Poor healthcare services are likely to result in poor nutritional and health outcomes including malnutrition and other diseases (17). Poor nutrition and health may in turn, negatively impact learning (cognition), absenteeism in school, educational attainment, employment, productivity and, ultimately, incomes with implication for food insecurity, poor dietary habits/intake, leading to poor health-related challenges including hormonal-related problems (18, 19).

There is vast geographical differences between the northern and southern sectors of Ghana in respect of the vegetation, diet and the population. Whiles northern Ghana is characterised mainly by grass and savanna vegetation with dry weather conditions, southern Ghana is largely forest and coastal (20). These geographical differences tend to influence agricultural practices, kinds of foods produced and diets. Available data indicates that over 50% of adolescent girls have low dietary diversity score (3.8+/−0.8) (21) even though consuming food from diverse sources largely offers protection against diet-related non-communicable diseases including all forms of malnutrition and hormonal imbalance (9). Reports indicate that adolescent girls' diets are dominated by energy-dense foods which may potentially have negative implication for their their hormonal health (22–24).

Hormonal disparities during adolescence have serious consequences for psychological well-being and reproductive health. Thus, understanding the influence of the interaction between SES and geography on hormonal levels is essential for designing tailored interventions that may improve adolescent health outcomes. The objective of the present study was to examine the regional variation and socioeconomic status associated with hormonal levels of adolescent girls in Northern and Greater Accra Regions.

## Materials and methods

### Study design

The study is part of a larger study that employed a qualitative approach, involving focus group discussion (FGDs), to collect in-depth data between June and October 2022. The approach offered insight into adolescent girls' knowledge, perceptions and experiences of hormonal imbalance. Thereafter, their blood samples were drawn and analysed to ascertain their serum hormonal levels across two distinct geographical locations.

### Study location and participant recruitment

The study was conducted in two districts in two administrative regions of Ghana—La Nkwantanan Municipality in Greater Accra Region (southern Ghana) and Tamale Metropolis in Northern Region (northern Ghana). In each district, 2 communities- one predominantly urban and the other predominantly rural—were selected, making a total of 4 communities. In each community, adolescent girls were sampled from upper primary (UP), Junior High School (JHS) and Senior High School (SHS). At the individual school level, a purposive sampling approach was used to select consented and assented adolescent girls who met the inclusion criteria and who were willing to share their experiences. Access to study participants was facilitated by authorities of the participating schools with written informed consent of parents/guardians.

### Study participants, eligibility criteria and sample size

The study targeted in-school adolescent girls aged 10 to 19 years. The inclusion criteria included adolescent girls who had experienced menarche and continued to have their menstrual periods at the time of data collection, those who were in UP, JHS and SHS, and those who assented to participate in the study together with their parents' consent. The exclusion criteria included adolescent girls who were in lower primary, were less than 10 years of age, had not experienced menarche and were not schooling at the time of the study.

In all, a total of 116 adolescent girls were recruited with 60 and 56 selected from Greater Accra and Northern Regions respectively. The sample of 116 participants in the study was arrived at based on the principle of data saturation in qualitative studies (25). In this study, saturation was achieved after the fourth FGD at each of the three educational levels when new information/data did not emerge from conducting additional FGDs. Hence, 4 FGDs, with an average of 9 participants per FGD, were conducted at each of the 3 educational levels, bringing the total to 12.

#### Study procedure and data collection

Familiarization visits to both study locations and their respective education directorates preceded data collection. Approval letters from Ghana Education Service to commence the study in the selected directorates were delivered to the appropriate officers for endorsement. Following this, the study team, with the assistance of the respective School Health Education Programme (SHEP) coordinators, met with authorities of the selected schools and parents/guardians of the participants and briefed them about the project and its objectives as well as sought their permission to conduct the study. Initial activities at the schools included booking appointment dates, times and venues for data collection. Fieldwork for the study commenced between June and October 2022 after an initial pre-test of the data collection instrument in a school located in a different location from the study areas. The pre-test facilitated the refinement of the instrument and ensured that only relevant questions were asked.

To ensure a fair representation of the different adolescent ages, the maximum variation sampling was applied in selecting participants from the 3 educational levels—UP, JHS and SHS—owing to their possible differential experiences of menstruation-related issues. At each of the educational levels, 4 FGDs were held with an average of 9 participants in each FGD. The FGDs were facilitated by a qualitative interviewer and assisted by a note-taker. Dietary and nutritional status data were collected by 4 nutritionists on the project while blood samples were collected by a qualified phlebotomist.

Data gathered from participants included their socio-demographic and economic characteristics such as age, age at menarche, menstrual-related experiences, dietary history and nutritional status (measured by body mass index and anaemia status) and blood samples. About 5 ml of blood was drawn from each participant and transported to the laboratory for processing. Blood samples were centrifuged at 2000 rpm for 10 min and serum stored at −20°C for further analysis. Additionally, data on parents (mothers and fathers/caregivers) socioeconomic variables—education, employment status and occupation (whether formal or informal sector worker)—were collected to assess the link between parental education and employment and occupation status and hormonal levels of participants in respect of androgen, estrogen and progesterone.

### Laboratory analysis

Commercially-available kits (*MyBioSource; San Diego, USA*) were used to determine the levels of hormones in the plasma using the Enzyme-linked immunosorbent assay (ELISA) according to manufacturer's instruction. Briefly, for estrogen (Cat No. MBS701834), precoated plates were allowed to warm to room temperature for about 30 min. Then 50 µl each of sample and estrogen HRP conjugate, and 50 µl of antibody were added and plates incubated for 2 h at 37°C. After incubation and washing, 50 µl each of substrate solution A and B were added for 15 min and reaction was stopped with 50 µl stop solution.

For progesterone (Cat No: MBS580029), a 20 µl sample was added to plates followed by 100 µl Progesterone Enzyme Conjugate, with 50 µl Biotin Conjugate. Plates were incubated for 60 min at room temperature. After washing thrice, 100 µl TMB was added and incubated for 15 min, and reaction was stopped with stop solution.

For androgen (Cat. No: MBS034696), 50 µl of sample and 50 µl of androgen HRP conjugate were added, and plates incubated for 60 min at 37°C. After incubation and washing, 50 µl each of chromogen solution A and B were added for 15 min and reaction was stopped with 50 ul stop solution. All the plates were read at 450 nm using a thermos multiscan plate reader.

### Data analysis

For the purpose of this paper, only relevant variables that addressed the objective of this paper were reported. The variables were participants' place of residence (northern or southern Ghana), age and educational level. Also of relevance were their parents/guardians' socio-demographic and economic characteristics: mother's and fathers' education, employment status and occupation (formal or informal worker). And finally, participants' hormonal assays were assessed.

#### Analysis of socio-demographic data

Data was analysed using R statistical software, version 4.3.0 (26) and GraphPad Prism, version 10.6.1 (892). Age was categorized into three: early adolescence (12–14 years), mid adolescence (15–16 years) and late adolescence (17–19 years). The Kruskal-Walis test was used to determine the differences in the hormonal levels of the three age-groups of adolescents across regions. The socio-economic statuses of parents (mothers and fathers) were categorized on the basis of (1) “educated” and “not educated” (formal education), (2) “employed” and “not employed” and (3) “formal sector worker” and “informal sector worker”.

The Mann–Whitney test was used to determine the differences between educated and not educated mothers. Residence or location was categorized as binary, and a generalized linear model was used to determine the hormonal levels associated with the respective locations. *P* values below 0.05 were considered statistically significant. Optical densities were converted to concentrations before analysis was done. We compared hormonal levels between adolescent girls from the north and the south. Differences in concentration between participants from the two regions were determined using the Mann–Whitney test.

## Results

### Demographic characteristics of participants

Participants from both sites had similar ages (*p* = 0.76) ranging between 12 and 19 years. Almost equal numbers of participants were drawn from the three levels of education: primary (31.9%), junior high school (33.6%), and senior high school (34.5%). Mean age at menarche, the occurrence of first menstruation, was 12.7 years. Northern participants (12.8 years) had a slightly higher age at menarche than Southern participants (12.6 years). About 55.2% of participants were in their late menarche (Delayed puberty). There were slightly more Christians (56.9%) than Muslims (43.1%). The data indicate that fewer mothers (64.7%) than fathers (68.1)had some level of education. Table 1 presents the relevant background charecteristics of participant.

**Table 1:** Demographic characteristics of study participants.

Variable	Level	North (*n* = 56)	South (*n* = 60)	Total (*n* = 116)
Mean Age (yrs)		15.5 (1.8)	15.4 (2.1)	15.5 (1.9)
Age Categories				
	12–14 yrs	21 (37.5)	20 (33.3)	41 (35.3)
	15–19 yrs	35 (62.5)	40 (66.7)	75 (64.7)
Mean (sd) age at Menarche		12.6 (1.3)	12.8 (1.4)	12.7 (1.3)
Father's Education^a^				
	Educated	40 (66.7)	39 (69.6)	79 (68.1)
	No formal education	7 (11.7)	10 (17.9)	17 (14.7)
	Unknown	13 (21.7)	7 (12.5)	20 (17.2)
Mother's Education^a^				
	Educated	39 (65.0)	36 (64.3)	75 (64.7)
	No formal education	21 (35.0)	20 (35.7)	41 (35.3)
Religion				
	Christian	37 (61.7)	29 (51.8)	66 (56.9)
	Muslim	23 (38.3)	27 (48.2)	50 (43.1)

a Education in this context means literate (formal education).

### Levels of androgen, estrogen and progesterone in adolescent girls in northern and southern Ghana

**Figure 1:**
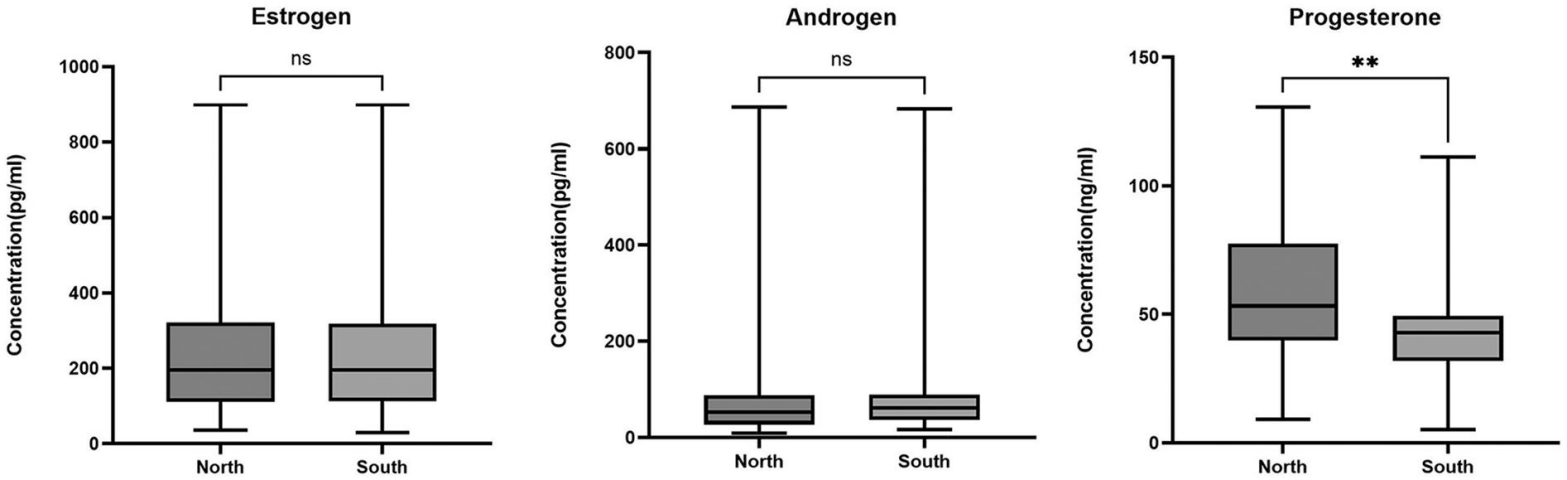
Hormonal levels in adolescent girls in northern and southern Ghana. The box plots represent hormonal levels between the north and south of Ghana. The middle black lines represent the median levels of hormones. *P*-values were determined using the Mann–Whitney test. *P < 0.05; **P < 0.001.

In both northern and southern locations, estrogen levels 195.5 (29.2–899.2.0) pg/ml were the highest followed by androgen 60.7 (8.2–687.3) pg/ml with progesterone being the lowest 46.0 (5.2–130.6) ng/ml. When the levels of plasma hormones were compared between the southern and northern adolescent girls, serum estrogen level measured was 165.8 (35.7–899.2) pg/ml in the north compared to 206.7 (29.2–899.2) pg/ml, in the south, *p* = 0.63. Androgen was 62.2 (13.9–683.6) pg/ml in the north and 60.2 (8.2–687.3) pg/ml in the south *p* = 0.81, and progesterone level was 49.5 (5.2–130.6) ng/ml in the north compared to 43.5 (9.3–108.3) ng/ml in the south *p* = 0.0019, Figure 1.

In this study, we categorized adolescence into early (12–14 years), middle (15–16 years), and late (17–19 years) stages. Hormonal levels were compared between the three stages in the girls in northern and southern Ghana independently. Generally, no statistically significant levels were observed between the three stages; however, a trend of high hormonal levels from early adolescence through to late adolescence was observed among girls in northern Ghana. A reverse trend was observed in southern Ghana where there were decreased hormonal levels from early adolescence through to late adolescence (Table 2).

**Table 2:** Hormonal levels at the different stages of adolescence.

Location	Hormone	Early adolescence	Middle adolescence	Late adolescence	Total
	Androgen	62.2 (37.5–79.5)	63.2 (38.1–105.8)	62.8 (31.8–82.5)	62.2 (36.2–84.8)
Northern Ghana	Estrogen	162.6 (109.3–356.0)	160.2 (111.2–245.5)	200.7 (100.7–433.3)	165.8 (110.8–316.6)
	Progesterone	43.4 (35.7–53.0)	52.7 (46.1–76.6)	54.3 (40.6–76.0)	49.5 (40.2–70.4)
	Androgen	71.6 (49.8–88.0)	66 (34.2–91.7)	50.8 (25.4–87.7)	60.2 (31.7–89.9)
Southern Ghana	Estrogen	225.3 (133.8–325.9)	209.4 (129.4–270.5)	170.8 (110.5–324.8)	206.7 (121–315)
	Progesterone	44.9 (38.8–54.6)	36.3 (27.3–41.5)	47.7 (35.9–66.0)	43.5 (32.5–54.8)

Table shows the median concentration with minimum and maximum concentrations.

### Parental educational attainment and hormonal levels in adolescent girls

**Figure 2:**
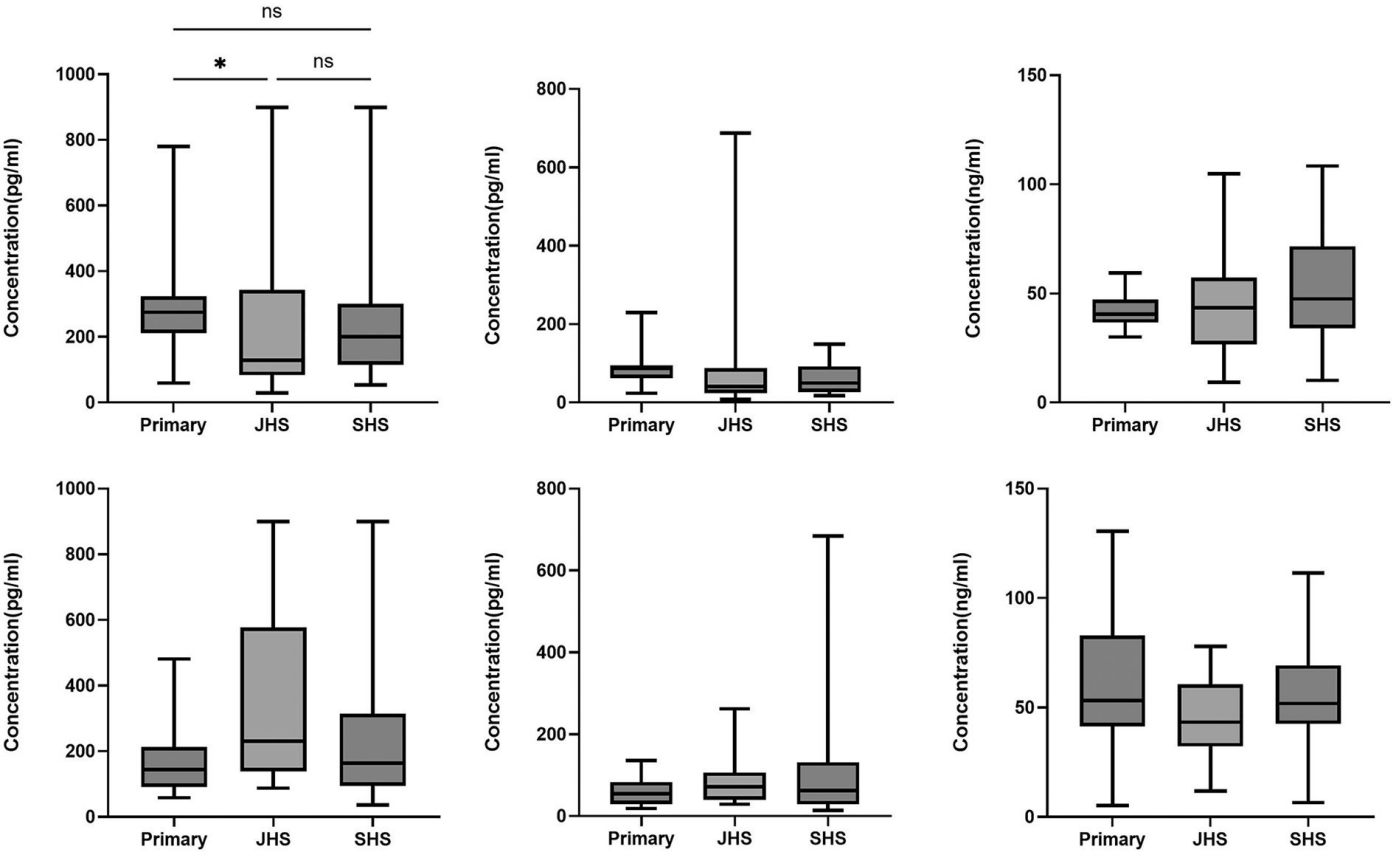
Levels of hormones among participants at different educational levels. The box plots represent hormonal levels based on the three educational stages, Primaryn the junior high school stage (JHS), and senior high school (SHS). The middle black lines represent the median levels of hormones *P*-values were determined using the Kruskal wails test. *P < 0.05; **P < 0.001.

When participants were grouped according to the three educational levels, no differences in the levels of the three hormones analyzed were observed among those in northern Ghana.In southern Ghana, however, the levels of androgen and estrogen were observed to be significantly higher in the primary school girls ( Figure 2 ).

### Parental employment/occupation and hormonal levels in adolescent girls

**Figure 3:**
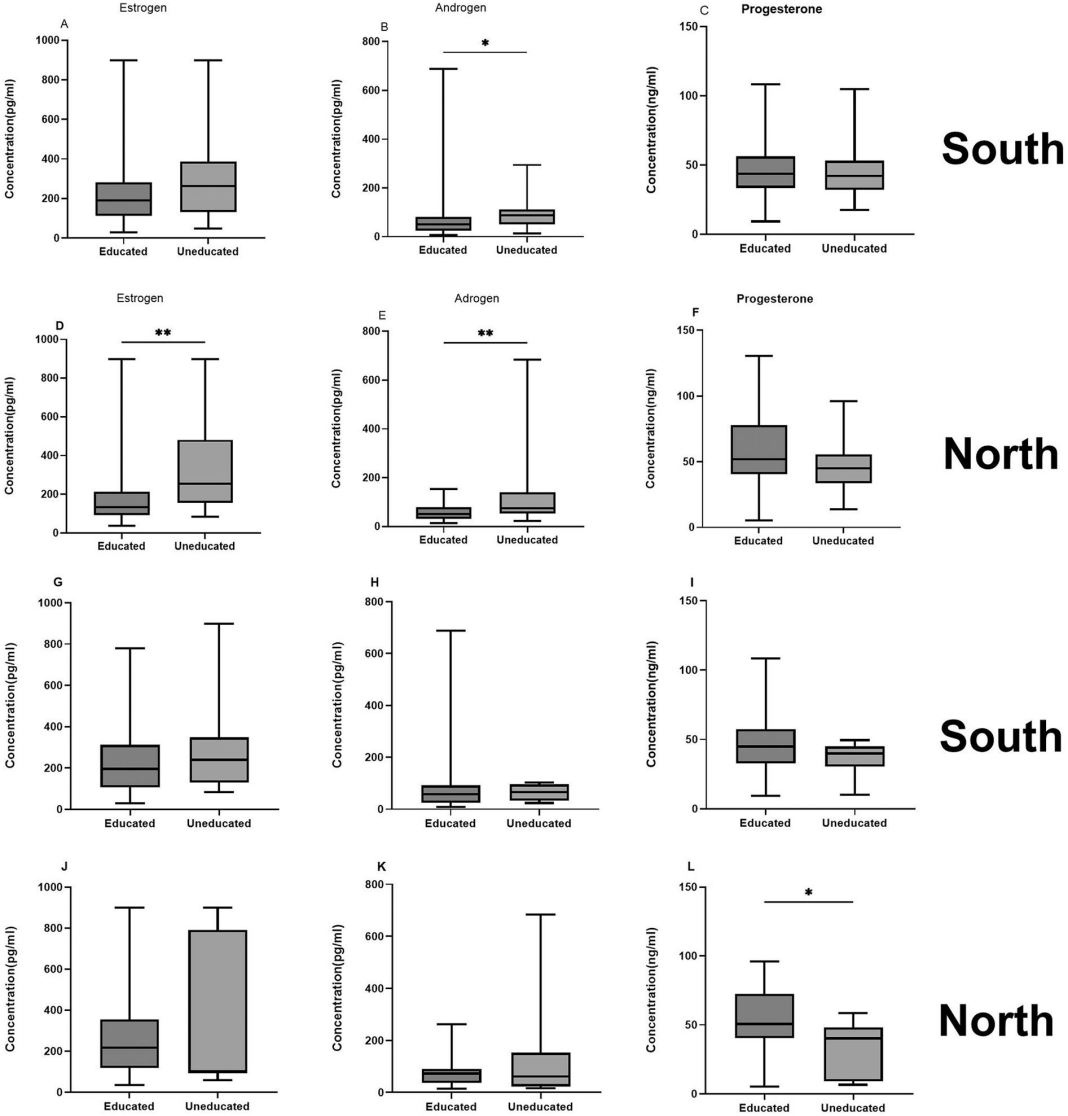
Hormonal levels in adolescent girls and their parental education. The box plots represent hormonal levels with the middle black lines representing the median levels. Parental educational levels were assessed ad a determinant of SES. These were categorized as having formal education or not, and graphs **(A–F)** represents mothers education for both locations for the three hormones and **(G–L)** represent fathers educational level. *P*-values were determined using the Mann Whitney test. *P < 0.05; **P < 0.001.

In a generalized linear model, participants whose mothers had no education were more likely to have higher plasma androgen [OR =  0.37, 95% CI = (0.21–0.68), *p* = 0.0011], and estrogen [OR = 0.37, 95% CI = (0.21–0.67), *p* = 0.0008] levels compared to those with educated mothers. When stratified according to location, participants in southern Ghana whose mothers had no formal education had significantly higher plasma levels of androgen (*p* = 0.017) and estrogen (*p* = 0.063) compared with those whose mothers were educated. A similar pattern was observed in the North where androgen (*p* = 0.009), and estrogen (*p* = 0.0012) plasma levels were higher in girls whose mothers had no education. No differences were observed in the plasma progesterone levels (Figure 3). There was also no difference in the proportion of fathers with and without formal education, *p* = 0.132. The educational status of 12% of the fathers was unknown. However the levels of progesterone in girls with educated fathers was higher than those with uneducated fathers [OR =  2.67, [95% CI (1.19–6.0), *p* = 0.018]. Generally 20.6% of the fathers had formal employment while only 10% of the mothers were formally employed. When stratified by location only 5% of the mothers had formal employment in the North while 11.5% were formally employed in the South. For the fathers, 18.3% held formal employment status in the north while 17.9% were formally employed in the south.

## Discussion

This study explored the regional variations and socioeconomic factors that influence the development of hormones, estrogen, progesterone, and androgen, in adolescent girls in northern and southern sectors of Ghana to ascertain influence of georgraphical/setoral differences. Our finding of higher estrogen levels in adolescent girls from both regions is indicative of the normal ongoing developmental processes. Estrogen is one of the steroid hormones associated with the development of female sexual characteristics. Estrogen also plays a vital role in puberty, mainly responsible for the development of the breast, changes in the body shape, and the overall maturation processes (27, 28). Thus, the generally high plasma estrogen levels corroborate other findings (29). The comparable levels of androgen across regions suggest a uniform sensitivity to androgens, which are produced in smaller amounts in females (30). The elevated progesterone levels among girls in northern Ghana might be influenced by dietary differences. In a study in the United Kingdom, it was found that zinc increased the amount of binding sites on progesterone receptors leading to increased plasma progesterone levels (31). Dietary pattern in Northern Ghana as reported in the literature (32) is predominantly cereal-based along with legumes, nuts and red meat. Nuts and red meat are rich in zinc (33) which could be the cause of the higher levels of plasma progesterone in the adolescent girls in the north. A probable factor for the lower progesterone levels in Southern Ghana may be the consumption of highly-processed food, which may interfere with normal hormonal functions. These highly-processed foods contain endocrine-disrupting chemicals which interfere with the biosynthesis of hormone, and metabolism by acting through the receptors for these hormones as antagonists (34, 35). Though we did not collect data on dietary patterns, it has been reported that diet has influence on hormone production (36).

The increasing trend of plasma hormonal levels observed in the north when adolescence was categorized into early, middle, and late stages reflects their physiological maturation through adolescence (37). The reverse trend observed in the south cannot easily be explained since in our thinking, the girls were following normal trajectory of pubertal growth with increasing hormonal levels. One reason that can be adduced for the decreasing hormonal trend in the south could be the consumption of highly-processed foods which may interfere with their normal hormonal levels. Since these participants attend schools located close to Accra, the national capital, the likelihood of consuming processed food is higher. There could also be potential differences in the dynamics or the regulatory mechanisms of these hormones (5, 38).

The higher levels of plasma androgen and estrogen observed among participants in primary schools in southern Ghana may suggest early pubertal development (39). This observation could be the product of stress and other socio-cultural influences on the girls, which is consistent with previous research that has shown that hormonal levels in adolescent girls are influenced by environmental and social factors (40, 41). Several studies have linked socio-economic status with various hormonal levels, stress and pubertal development (12, 15, 40, 42). In this study, we defined high socioeconomic status as having completed/attained a mid-level or higher level of education and the parental occupation, whether they had formal employment or not. The ability of parents with good socio-economic status—in this case, education and formal employment—to give their adolescent girls better care motivated us to find out whether the educational levels and occupation of parents of adolescent girls in our study could influence the levels of the hormones studied. Our data suggests that girls whose mothers have no formal education are more likely to have elevated androgen and estrogen levels. Conversely, those with educated fathers had higher levels of progesterone. This may suggest parents influencing the food choices at home thereby (43) providing their adolescent daughters natural and traditional foods which are better for the girls. Thus, there may be hormonal stability compared to the educated mothers. Though we do not have data on processed food consumption by the girls, we believe the low levels in children with educated parents could result from the consumption of highly-processed foods containing endocrine-disrupting factors (EDC) which tend to disrupt hormonal synthesis (34, 35). Some of these EDCs are plastiscs and plasticizers which contaminate the food during processing and storage (34). A study conducted in Ghana found that ultra-processed food accounted for about 30% of all processed food in the urban centres (44). In another study in Malaysia, children of educated mothers were found to have higher fast food intake compared to children of uneducated mothers (45). Fast-food, which is highly processed and common, contains-endocrine disrupting chemicals which could affect the synthesis and production of hormones. The similar trend of children of mothers with no formal education with elevated levels of androgen and estrogen indicates some consistency and highlights the global impact of socio-economic status and change in diet on hormonal status. This issue underscores the importance of addressing socio-economic disparities in the health of adolescent girls by helping vulnerable populations.

## Limitations of the study

Though this current paper does not include information on adolescent girls' dietary intake/behaviour, which is being reported in another paper, its non-inclusion is a limitation to this paper; studies have found mothers' socio-economic status to influence their dietary choices (15, 44, 45). Again, restricting the study to only two administrative regions and employing a non-probability sampling approach to selection of only 116 adolescent girls may have introduced some sampling bias in addition to making the study findings not to be nationally representative enough. In spite of this weakness, the findings from this study gives useful information on the hormonal profiles of adolescent girls in the two regions. Furthermore, the lack of longitudinal data on homornal levels posses another challenge which must be investigated in future studies.

## Conclusions

In summary, the study provides useful information on hormonal profiles of adolescent girls in northern and southern Ghana and highlights the link between maternal socio-economic status and hormonal health in adolescent girls. This study also highlights the complex relationship between geographical location and hormonal levels in adolescent girls. This study calls for measures to address practices that cause the differences between hormonal levels in adolescent girls with educated and uneducated mothers may have implications for their health, fertility and well-being.

## Data Availability

The original contributions presented in the study will be made available upon reasonable request, further inquiries can be directed to the corresponding authors.
